# Anomalous Interarterial Right Coronary Artery: Approach to a High-Risk Course

**DOI:** 10.7759/cureus.29991

**Published:** 2022-10-06

**Authors:** Pradnya Brijmohan Bhattad, Anil Jha, Luigi Pacifico, Eddison Ramsaran

**Affiliations:** 1 Cardiovascular Medicine, Saint Vincent Hospital, University of Massachusetts Chan Medical School, Worcester, USA

**Keywords:** cardiac sudden death, congenital coronary anomalies, coronary angiography, interarterial course, anomalous origin of right coronary artery

## Abstract

Congenital coronary anomalies can be an incidental finding in the adult population. Implications of an anomalous coronary artery vary depending on its course and the anomaly. An interarterial course of an anomalous coronary artery is considered malignant with a high risk of sudden cardiac death. The presentation of the interarterial course of an anomalous coronary artery is variable. We report a rare case of an anomalous origin of a right coronary artery presenting with vague symptoms without any evidence of inducible ischemia. Given the rarity of an anomalous interarterial right coronary artery, the implications of this congenital anomaly on physical activity, treatment options including surgical correction, and estimating the risk of sudden cardiac death are difficult based on currently available data.

## Introduction

A coronary artery originating from the opposite sinus of Valsalva is an uncommon congenital coronary anomaly with the risk of sudden cardiac death or ischemia. Anomalous origin of the left circumflex coronary artery (LCx) from the right sinus of Valsalva is much more common compared to an anomalous origin of the right coronary artery (RCA) originating from the left sinus which has a prevalence rate between 0.026% and 0.25% [1,2]. The clinical presentation of an anomalous origin of RCA is variable. Anomalous coronary arteries can arise without any evidence of structural heart disease [2,3].

## Case presentation

A 42-year-old male with a history of dyslipidemia but no other significant medical history underwent ongoing evaluation for complaints of vague substernal chest discomfort. He described intermittent episodes of sharp non-radiating substernal chest discomfort with occasional dizziness but no other associated symptoms. He reported these episodes lasting for a few minutes without any clear precipitating or relieving factors. He complained of these ongoing symptoms for the preceding two years occurring intermittently, with a frequency of three to four times per week. He denied any exertional symptoms or any association of the symptoms with physical activity, rest, or sleep. A complete review of systems was otherwise unremarkable. He was hemodynamically stable. A complete physical examination was normal without any evidence of fluid overload.

**Figure 1:**
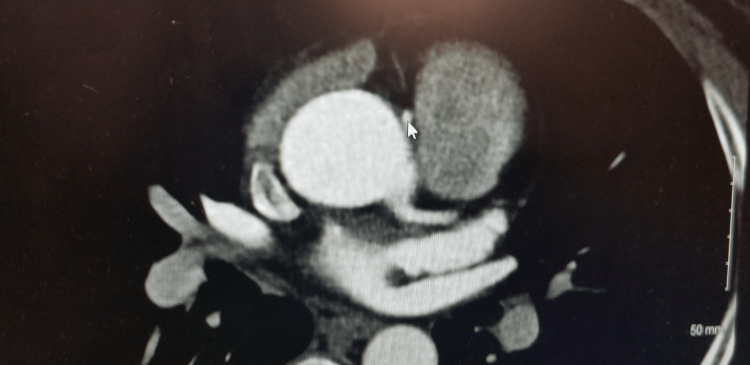
Interarterial course of the right coronary artery as seen on computed tomography angiogram.

His complete blood cell count, chemistry panel, electrolytes, thyroid function studies, cardiac biomarkers and enzymes, inflammatory markers such as erythrocyte sedimentation rate, and C-reactive protein were normal. An electrocardiogram (ECG) showed normal sinus rhythm. A regadenoson pharmacologic stress test was normal. A dobutamine stress echocardiogram was normal. He reported exercising regularly on a treadmill without any symptoms. He had no personal or family history of sudden cardiac death. He underwent a computed tomography angiogram (CTA) of the chest which showed no evidence of pulmonary embolism but revealed an incidental finding of an anomalous origin of the RCA arising from the left coronary cusp with an interarterial course (Figure 1).

Given his vague symptoms and the incidental finding of anomalous origin of RCA on CTA, he underwent cardiac catheterization which showed angiographically normal coronary arteries with anomalous origin of the RCA arising from the left coronary cusp (Videos 1-4).

**Video 1:** Left coronary system on coronary angiogram.

**Video 2:** Coronary angiogram of the left coronary artery in the same patient with an anomalous right coronary artery.

**Video 3:** Coronary angiogram demonstrating an anomalous right coronary artery arising from the left coronary cusp.

**Video 4:** Coronary angiogram shows an anomalous right coronary artery arising from the left.

He underwent rapid right atrial pacing at a rate of 150/minute during the cardiac catheterization during which there was no evidence of ischemia clinically or on ECG. A transthoracic echocardiogram revealed mildly reduced left ventricular systolic function with a left ventricular ejection fraction of 50% with no segmental wall motion abnormalities. A Holter monitor did not show any evidence of any significant arrhythmias.

He was evaluated by cardiothoracic surgery and after shared decision-making, he preferred a conservative approach without any surgical intervention. He will be followed up closely as an outpatient.

## Discussion

The course of an anomalous RCA is variable. Usually, an anomalous RCA or left main coronary artery originating from the opposite sinus may have one of the following four courses: (i) anterior to the pulmonary artery, (ii) posterior to the aorta, (iii) subpulmonic or septal course, and (iv) interarterial course. Usually, the pulmonary artery is anterior and superior to the aortic origin and somewhat in the leftward direction [2,4,5].

The interarterial course of the anomalous coronary is commonly associated with exercise-induced myocardial ischemia with a risk of ventricular arrhythmia and sudden cardiac death, especially during exercise [1,3]. In an interarterial course of an anomalous coronary artery, the origin is usually sharply angled and oblique leading to a slit-like ostium of the vessel which has the potential to get stretched during exercise with aortic distention, which allows further narrowing of the ostium leading to exercise-induced ischemia [2,4,6]. The interarterial course of an anomalous coronary artery also has a risk for torsion, and this course is considered the most common course of an anomalous RCA [3-6].

The other courses of an anomalous coronary artery have been described to be less likely associated with sudden cardiac death in the current literature. To define the course of an anomalous coronary artery, a left ventriculogram or an aortogram in a right anterior oblique view is usually performed, and computed tomography or magnetic resonance imaging is generally used to confirm these findings [4,6,7].

The risk of sudden cardiac death or exercise-induced ischemic symptoms such as chest pain, syncope, and presyncope usually presents before the age of 25 in the case of an anomalous RCA. A stress test is considered insensitive to predict the risk of sudden cardiac death or to diagnose exercise-related ischemia from an anomalous coronary artery [2,4,5]. Surgical management has been indicated in patients who have had an anomalous interarterial RCA with documented evidence of exercise or stress-induced ischemia [2,4,8]. The current guidelines from the American College of Cardiology suggest that the slit-like orifice or a long intramural course of the anomalous artery is a surgical indication rather than the interarterial course. The finding of an anomalous interarterial RCA or left main coronary artery in a younger individual aged less than 35 years may be considered for surgical unroofing of the coronary intramural course and relocation after thorough evaluation [3-5,8].

## Conclusions

Anomalous origin of the RCA from the left coronary cusp with an interarterial course between the aorta and pulmonary artery is a rare congenital coronary anomaly. It does have a potential for exercise-induced ischemia which may lead to sudden cardiac death, especially in the younger population. The current literature suggests that surgical correction of an anomalous RCA arising from the left sinus with an interarterial course may be considered if the patient has symptoms that are attributable to ischemia or any documented evidence of ischemia, family history of sudden cardiac death, or inducible ischemia. Detailed coronary anatomy should be studied with multimodality imaging studies, and the course of the anomalous coronary artery must be studied for medical decision-making. Physiologic testing may be sometimes necessary to evaluate for inducible ischemia. There are currently no specific guidelines to approach a high-risk course of a congenital coronary anomaly such as an anomalous RCA from the left coronary cusp with an interarterial course and more research is indicated in this area.

## References

[1] Lee HJ, Hong YJ, Kim HY (2012). Anomalous origin of the right coronary artery from the left coronary sinus with an interarterial course: subtypes and clinical importance. Radiology.

[2] Cheezum MK, Liberthson RR, Shah NR, Villines TC, O'Gara PT, Landzberg MJ, Blankstein R (2017). Anomalous aortic origin of a coronary artery from the inappropriate sinus of Valsalva. J Am Coll Cardiol.

[3] Ferreira AF, Rosemberg S, Oliveira DS, Araujo-Filho JA, Nomura CH (2019). Anomalous origin of coronary arteries with an interarterial course: pictorial essay. Radiol Bras.

[4] Peñalver JM, Mosca RS, Weitz D, Phoon CK (2012). Anomalous aortic origin of coronary arteries from the opposite sinus: a critical appraisal of risk. BMC Cardiovasc Disord.

[5] Mirchandani S, Phoon CK (2005). Management of anomalous coronary arteries from the contralateral sinus. Int J Cardiol.

[6] Angelini P, Velasco JA, Flamm S (2002). Coronary anomalies: incidence, pathophysiology, and clinical relevance. Circulation.

[7] Taylor AJ, Byers JP, Cheitlin MD, Virmani R (1997). Anomalous right or left coronary artery from the contralateral coronary sinus: "high-risk" abnormalities in the initial coronary artery course and heterogeneous clinical outcomes. Am Heart J.

[8] Lee BY (2009). Anomalous right coronary artery from the left coronary sinus with an interarterial course: is it really dangerous?. Korean Circ J.

